# Successful separation of xypho-omphalopagus conjoined twins with extrauterine twin-twin transfusion syndrome: a case report

**DOI:** 10.3325/cmj.2019.60.301

**Published:** 2019-08

**Authors:** Ruža Grizelj, Nada Sindičić Dessardo, Krešimir Bulić, Tomislav Luetić, Danko Mikulić, Anko Antabak, Ivica Sjekavica, Ana Marija Alduk, Sanja Konosić, Karolina Režek Tomašić, Tomislav Ćaleta, Sanja Pleško, Dalibor Šarić, Jurica Vuković

**Affiliations:** 1 Department of Pediatrics, University Hospital Center Zagreb, Zagreb, Croatia; 2 University of Zagreb School of Medicine, Zagreb, Croatia; 3 Department of Surgery, University Hospital Center Zagreb, Zagreb, Croatia; 4 Department of Surgery, University Hospital Merkur, Zagreb, Croatia; 5 Department of Diagnostic and Interventional Radiology, University Hospital Center Zagreb, Zagreb, Croatia; 6 Department of Anesthesiology, University Hospital Center Zagreb, Zagreb, Croatia; 7 Department of Clinical and Molecular Microbiology, University Hospital Center Zagreb, Zagreb, Croatia

## Abstract

Conjoined twining is a rare medical phenomenon, with an overall prevalence of 1.47 per 100 000 births. This report describes a successful separation of xypho-omphalopagus conjoined twins complicated by unbalanced blood shunting through the porto-systemic anastomoses within the shared liver parenchyma. Significant extrauterine twin-twin transfusion syndrome caused by unbalanced shunting is an extremely rare, and probably under-recognized, hemodynamic complication in conjoined twins necessitating urgent separation. Progressive deterioration with a poor outcome can be prevented if the condition is recognized in a timely manner.

Conjoined twinning is a unique complication of monochorionic pregnancy, with a total prevalence of 1.47 per 100 000 births (1). Seventy percent of conjoined twins (CT) are female, and the majority are either stillborn or die early in life (2,3). According to Croatian Birth Registry, the incidence of liveborn CT in the period of 12 years is 1.24 per 100 000 live births, and CT represents 0.05% of all liveborn twin sets. Considering these data and the heterogeneity of CT anatomy and physiology it is difficult to define a standard of care. The occurrence of a serious illness in one of the twins or specific physiologic conditions as an unbalanced circulation represent complications that may require an early separation attempt, which carries a significantly increased mortality risk. To date, only a few cases of extrauterine twin-twin transfusion syndrome (TTTS) in CT have been reported (4,5). We present a case of successfully separated xypho-omphalopagus CT with extrauterine TTTS due to significant unbalanced crossover circulation.

## Case report

**Figure 1:**
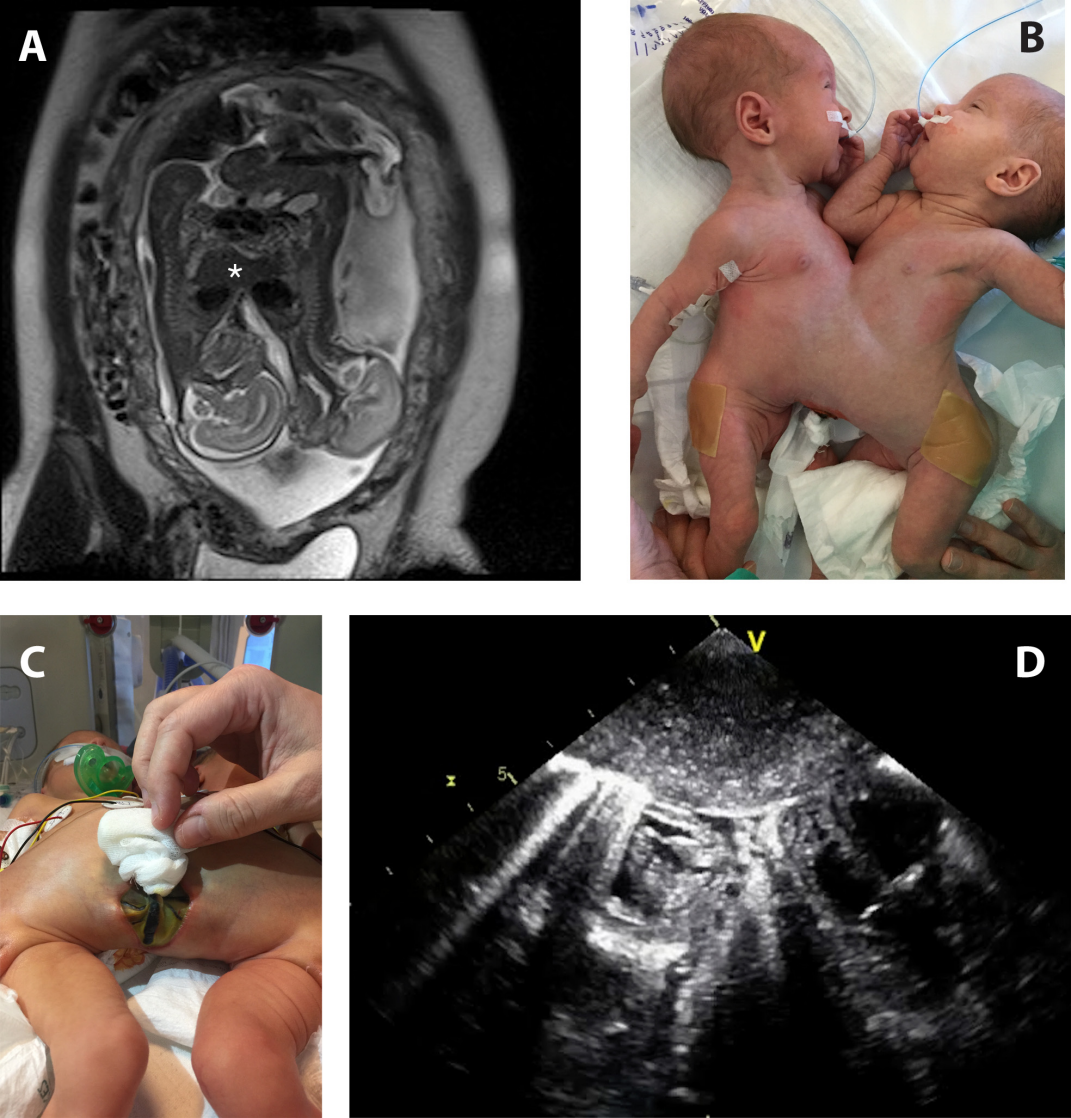
(**A**) A fetal T2 coronal magnetic resonance image (MRI) of conjoined omphalopagus twins at 21 weeks of gestation. A single liver is shared between both fetuses (*). Each fetus has a separate heart, stomach, kidneys, and urinary bladder. (**B**) and (**C**) Photographs of the twins. (**D**) A two-dimensional echocardiography image before separation showing separate hearts and pericardium. The heart apexes pointing toward each other (heart of the twin B is rotated to the right with the apex pointing toward the apex of twin A, which is in the normal left position). The distance between the apexes is 5-6 mm. The hearts were separated with the lower part of the sternum.

Symmetrical female xypho-omphalopagus CT were born by elective C-section at 33^2/7^ weeks gestation to a healthy 24-year-old gravida 3, para 2 woman. A monochorionic monoamniotic conjoined twin pregnancy was discovered on sonography at 11 weeks gestational age (GA) and confirmed as omphalopagus by a fetal magnetic resonance imaging at 21 weeks GA (Figure 1A). There was no evidence of growth discordance or hydrops in either of the twins. At birth, the twins were found to be joined ventrally from the xyphoid process to the umbilicus. The presence of a small omphalocele as a part of the joining bridge, with a single umbilical cord attached on the inferior aspect of the bridge, was noted (Figure 1B). The twins’ combined birthweight was 3.8 kg. The babies were designated as “twin A” and “twin B.”

Echocardiogram revealed two structurally normal and separated hearts (Figure 1C). An abdominal ultrasound confirmed the presence of a liver parenchymal bridge with one gallbladder (twin B), no dilatation of the biliary tracts, and the presence of two distinct hepatic hila with two separate venous portal systems with good hepatopetal flow. Doppler ultrasound confirmed hepatic venous drainage into the inferior vena cava and right atrium of each twin. No aberrant vascular connections were detected within the common liver parenchymal bridge. Each twin had two normal kidneys and the spleen.

Both developed respiratory distress syndrome shortly after birth, received surfactant, and were mechanically ventilated for 72 hours, with subsequent non-invasive respiratory support for additional four days (twin A) and 13 days (twin B). Enteral feeding with mother’s milk was initiated at day 4. At day 12, the twin A developed clinical and radiologic signs of necrotizing enterocolitis (NEC). Feedings were discontinued in both twins, and antibiotics were started with good treatment response.

**Figure 2:**
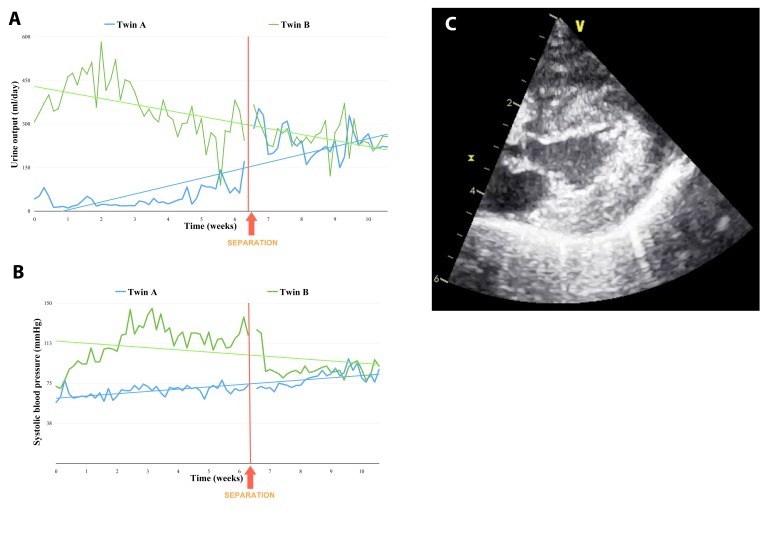
Daily urine output (**A**) and systolic blood pressure (**B**) of twin A (blue line) and twin B (green line) after birth. The arrow and red line indicates the time of separation. Trendlines are superimposed on the charts to reveal the overall direction of the data. (**C)** A two-dimensional echocardiogram showing apparent left ventricular hypertrophy in twin B.

Oliguria (42 mL/day) in twin A and polyuria (308 mL/day) with rapidly progressive hypertension in twin B were noted immediately at birth and persisted until the end of 5th week, when the physiologic situation reversed; diuresis gradually began to increase in twin A and concomitantly decreased in twin B (Figure 2A). Serum electrolytes, creatinine, and blood urea nitrogen were regularly obtained, and all the results in both twins were repeatedly exactly the same and within the reference values. Twenty-four hour creatinine clearances at the 30th day of life were 32 and 54 mL/min/1.73m^2^ for twin A and B, respectively.

Echocardiogram revealed left ventricular hypertrophy in severely hypertensive (blood pressure 145/68 mm Hg) and polyuric twin B, accompanied by a substantial difference in calculated cardiac output between the twins: 0.372 L/min in twin A vs 1.031 L/min in twin B (Figure 2B). Cardiac biomarkers, high-sensitivity cardiac troponin T (hs-cTnT) and N-terminal pro-brain natriuretic peptide (NT-proBNP), were respectively 52 ng/L (reference range <14 ng/L) and 3711 ng/L (reference range <300 ng/L) in both twins. Over the same period, the changes in systolic blood pressure showed a similar but less pronounced pattern; ie, upward in twin A and downward in twin B (Figure 2A).

**Figure 3:**
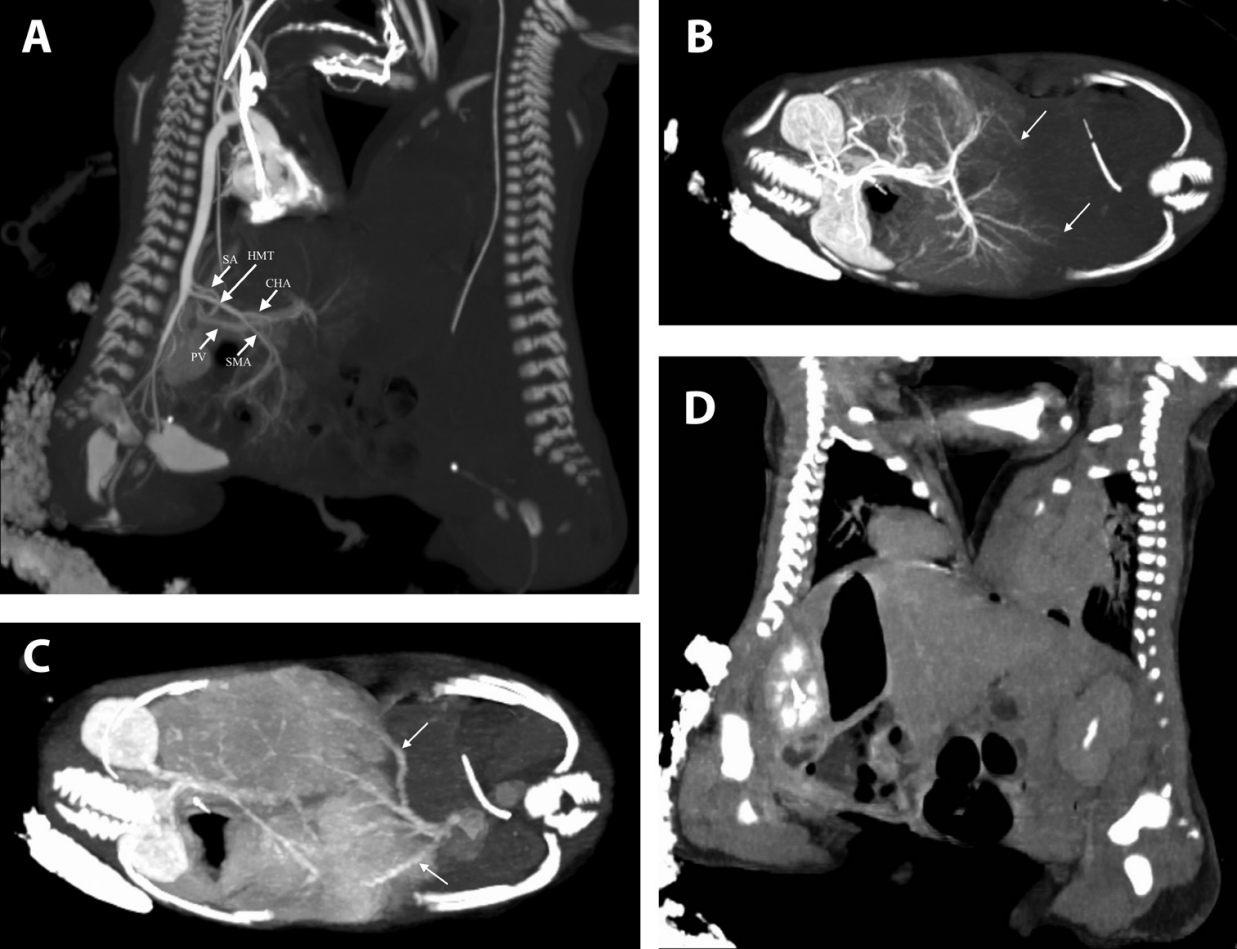
Dynamic contrast-enhanced computed tomography with multi-projection volume reconstruction images. (**A**) A sagittal image of the arterial phase showing the separate origin of the splenic artery (SA), a common origin of common hepatic artery (CHA), and the superior mesenteric artery (SMA) representing the hepato-mesenteric trunk (HMT), and normal anatomy of twin A's portal vein (PV). (**B**) An axial image of the arterial phase showing enhanced hepatic vein branches in twin B (arrows) communicating with twin A's portal branches, representing portosystemic crossover shunts. (**C**) A portosystemic crossover shunt is confirmed by pronounced opacification of hepatic veins in twin B (arrows) on an axial image of the venous phase. (**D**) A delayed image showing persistent nephrogram in twin A and normal pyelographic phase with a contrast in the renal pelvis in twin B.

Dynamic contrast-enhanced computed tomography of the liver was performed on the 16th day of life to evaluate the magnitude of cross-circulation between the twins. Contrast medium was administered to twin A only. In the arterial phase, the anatomy of the arterial vasculature was delineated in twin A (Figure 3A). The usual celiac trunk was not identified. The splenic artery and the hepato-mesenteric trunk (common hepatic artery and superior mesenteric artery) were independently arising from the abdominal aorta. The portal vein of twin A was also demonstrated. In the part of the liver belonging to twin B, multiple enhanced hepatic vein branches communicating with twin A’s portal branches, representing porto-systemic shunts, were observed (Figure 3B). The hepatic veins of twin B were clearly delineated in the venous phase, confirming the presence of cross-circulation through fused liver parenchyma (Figure 3C). In the late urographic phase, when the contrast agent appeared in the renal pelvis and urinary bladder of twin B, it was still persisting in twin A’s collecting system (Figure 3D). The pancreases were not well visualized, and it was not possible to determine the extension of the shared parts of the small bowel. The warning signs of imminent cardiac failure in twin B prompted us to proceed with the operation at the age of 45 days and the combined body weight of 4.7 kg.

### Surgical procedures

**Figure 4:**
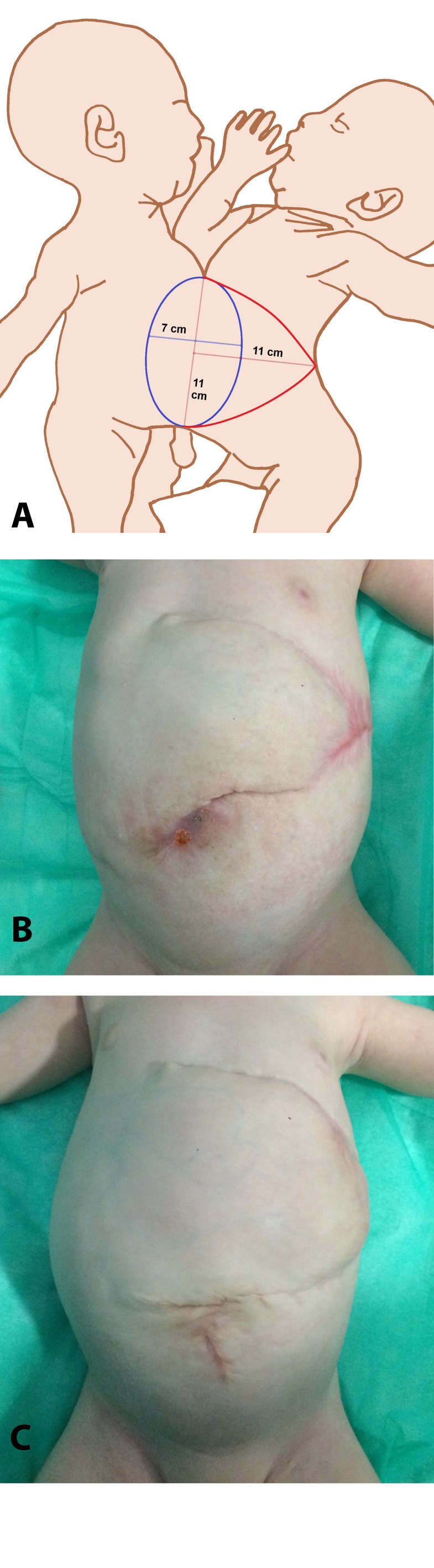
(**A**) A drawing of the estimated abdominal wall defect and incision placement. Abdominal wall reconstruction in twin A (**B**) and twin B (**C**) four months after the primary operation.

**Figure 5:**
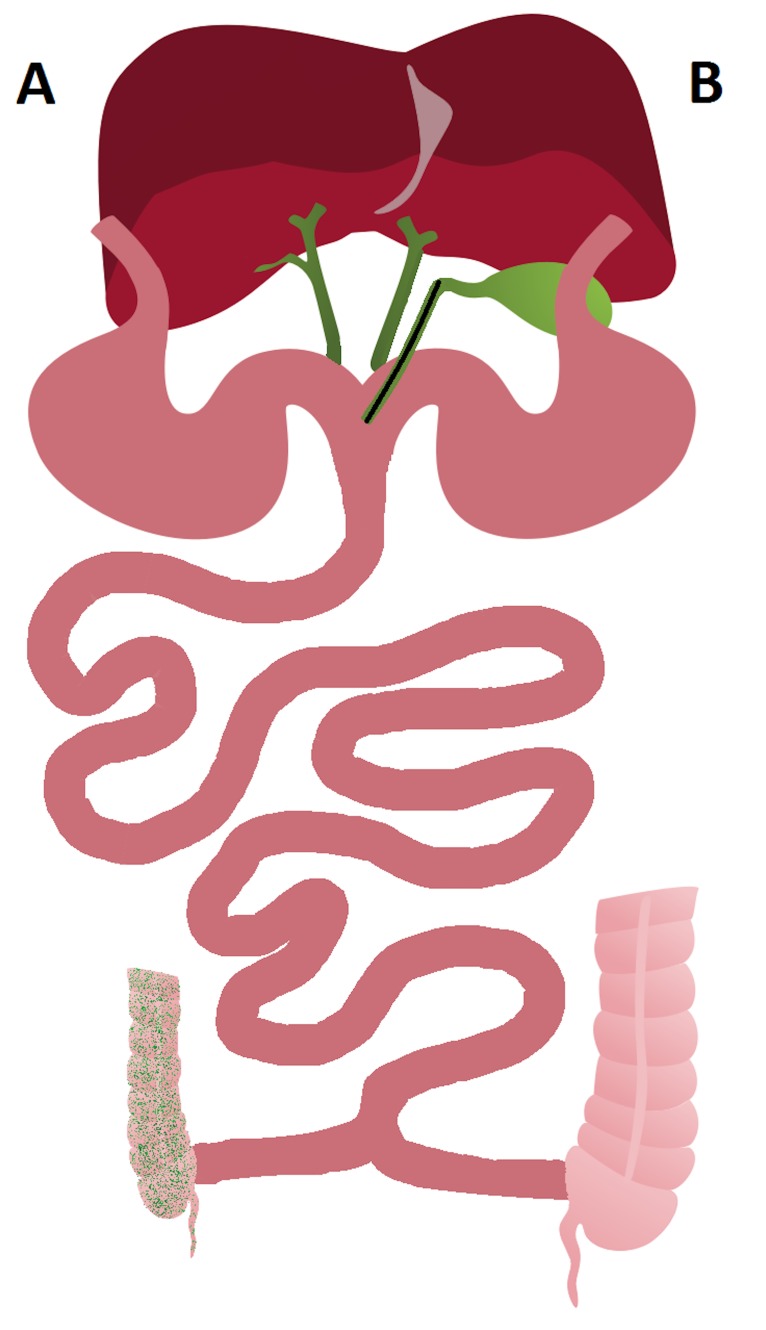
A drawing demonstrating the shared anatomy of the common gastrointestinal tract. The upper gastrointestinal tract joined in the duodenal region and separated at the level of the Meckel’s point into two separate terminal ileal segments and two colons. Beyond the shared segment, each twin had 40 cm of small bowel prior to the ileocecal valve. The large intestines were separate. Note a single gallbladder on twin B’s side of the liver with an obliterated cystic duct connected with the duodenum, and twin A’s colon affected with necrotizing enterocolitis.

The operation duration was 15 h, without any intraoperative complications. It started with raising of a large triangular fasciocutaneous flap, whose base extended from the upper to the lower border of joined abdominal wall connection, whereas the tip extended past the posterior axillary line on the opposite twin (Figure 4A). While elevating the flap, we noticed fully developed muscular components of the abdominal walls. The joint abdominal cavity was entered through the fascial connection between the twins’ abdominal walls, which resembled a white line, and joined costal cartilages and the xyphoid process were separated without opening the pericardial sacs. Intraabdominal exploration revealed two mirror image livers joined by a bridge of liver tissue that was approximately 6 cm long and 5 cm thick (Figure 5). The results of preoperative imaging were confirmed, with each liver having a separate hilum and a separate retrohepatic vena cava. A single gallbladder was attached to the twin B's liver with an obliterated cystic duct. Two extrahepatic bile ducts were visible. Twins shared a substantial portion of the upper gastrointestinal tract, which was well vascularized with vascular pedicles originating from both twins. Twin A's distal ileum and colon revealed multiple residua of past NEC. The division of the small bowel was designed to provide each infant with enough small bowel to avoid postoperative short-gut syndrome: twin A's duodenum was left in continuity with the shared upper small bowel, and her terminal ileum was opened as terminal ileostomy. The severity of colon destruction that occurred during and post NEC was assessed during operation and decision was made to perform subtotal colectomy leaving only the sigmoid and rectum. Twin B's duodenum was anastomosed to her distal small bowel, which was in continuity with her large bowel. The liver was then transected along the median line between the two portal triads using bipolar cautery with hemoclips and ligatures securing major crossing vessels and bile ducts. The single gallbladder was excised.

The twins were then turned 180 degrees thus exposing the posterior side. Another triangular flap was raised and the fascial connection was divided, thus completing the separation. A prosthetic mesh was used to reconstruct the musculo-fascial layer of the abdominal wall defects, while the soft tissue cover was provided by the triangular fasciocutaneous flaps.

In twin B, a complete defect closure was achieved at the time of the primary operation, with one reduction of the prosthetic mesh 3 months later (Figure 4B). In twin A, a complete closure was not possible at the time of the primary operation owing to a relatively larger portion of the liver received and intestinal edema. The abdominal wall defect was closed with a prosthetic mesh and covered with the triangular flap in the upper half, leaving about a half of the mesh exposed. The flap donor site defect was closed directly, while the remaining exposed mesh was covered in several additional procedures when the intestinal edema subsided and the size of the mesh was reduced. Bowel continuity was restored 50 days after the primary operation, and the abdominal wall was completely reconstructed 3 months later (Figure 4C).

After the separation, the urine output in twin A immediately rose to 290-350 mL/day (4.5-5.5 mL/kg/h). Functional kidney tests in both twins were within the reference range, and twenty-four hours creatinine clearances were 114 and 117 mL/min/1.73m^2^, respectively.

In twin B, milrinone treatment was started following operation and continued until the blood pressure steadily dropped to the reference values. Gradual regression of myocardial hypertrophy was observed, leading to complete normalization of echocardiographic appearance. High-sensitivity cardiac troponin values returned to the reference levels, while NT-proBNP (385 ng/mL) was still slightly elevated before discharge. Echocardiogram and both cardiac markers (hs-cTnT, NT-proBNP) were within the reference range in twin A. In both twins no significant alteration of bilirubin levels and liver function tests was observed. They ultimately achieved full enteral feeding and appropriate weight gain.

## Discussion

CT incidence in Croatia is within the range observed in Western populations (1.02-1.34 per 100 000 births) (1). There were six liveborn sets of CT (five female) during the last twelve years in Croatia (four toracopagi, and two omphalopagi – one of them minimally conjoined). Omphalopagus type comprise 5% of all CT and they are usually fused ventrally in the umbilical area, commonly involving the lower thorax but never involving the heart (1,6). Liver fusion is found in approximately 80%, with a common biliary tree in 20% of cases (7). In up to one-third, the intestines usually join at jejunal level with an ileal divergence close to the ileo-cecal valves (8).

Despite the fact that CT often have vascular shunts and cross-circulation, to the best of our knowledge only a few cases of hemodynamically significant unbalanced circulatory shunting have been reported so far (4,5). Vital functions monitoring and day-to-day clinical evaluation of our patients suggested the existence of a continuous unidirectional shunt between the twins, presumably hemodynamically similar to a prenatal TTTS, a serious complication caused by unbalanced blood shunting between twins with monochorionic placentation before birth. Prenatal unidirectional flow from one twin to the other causes volume depletion in the donor twin, with subsequent oliguria, oligohydramnios, and poor fetal growth, and volume overload in the recipient twin, with polyuria, polyhydramnios, and occasionally heart failure (9). A considerable degree of cross-circulation through the shared liver parenchyma in our case led to identical hemodynamic imbalance and development of TTTS syndrome after birth, ie, oligoanuria in twin A and polyuria and hypertensive cardiomyopathy in twin B. The condition is probably under-recognized and may lead to unfavorable outcomes (10). We believe that unidirectional blood shunting from twin A to twin B through the common liver before operation caused reduced systemic perfusion, leading to splanchnic hypoperfusion, intestinal ischemia, and ultimately NEC in twin A (11,12). Despite common belief that NEC is an unexpected and rare event in conjoined twins, a number of reports show that NEC occurrence in omphalopagus CT is higher than expected for GA (4,13-15). It is prudent to assume that the unique anatomy of conjoined twins might be the basis for dynamic and unexpected changes in shared vascular beds, leading to unbalanced circulation. Such an impaired blood flow could easily rank among the other typical NEC-related risk factors (hypoxia, ischemia, blood transfusion, umbilical vessels catheterization, etc). In our case, immaturity of the intestinal tract was an additional factor that could have contributed to the emergence of NEC in twin A.

The appearance of both nephrographic and pyelographic phases on delayed images in non-injected twin B confirmed considerable shunting between the twins. On the other hand, a persistent nephrogram in twin A probably indicated a generalized reduction of her glomerular filtration rate as a consequence of altered glomerular hemodynamics. Preoperative oliguria in the underperfused twin reversed immediately after separation, similar to earlier published cases (4,5). Since serum creatinine, as well as other hematologic and biochemical findings, were similar in each twin preoperatively, we assume that laboratory tests can be misleading when there is a substantial degree of cross-circulation. In such a situation, individual renal function can be assessed by careful monitoring of urine output and obtaining serial urine creatinine clearance. Gradual reversal of hypertension and hypertrophic cardiomyopathy in overperfused twin B were also observed. In the presence of severe disparity of renal function, early separation is indicated to prevent renal failure and compensatory cardiac failure (4). Adequate soft tissue reconstruction of the abdominal wall defects resulting from the separation is of paramount importance in achieving an esthetically pleasing outcome and preventing postoperative infections. An ideal reconstruction following such a separation should restore the musculo-fascial layer and adequate skin/soft tissue cover. The most successful reconstructions have been achieved through the use of tissue expansion before the separation (16,17). Unfortunately, owing to the relative urgency for separation in our patients, this was not a viable option. Based on the preoperative computed tomography scan, we estimated that the separation would result in an 11-by-7 cm full thickness defect of the abdominal wall, representing a defect of almost one third of the entire trunk circumference. We used a prosthetic mesh to reconstruct the musculo-fascial layer, while soft tissue cover was provided by creating large triangular fasciocutaneous flaps on each twin. We calculated that the flap length of 11 cm would directly close all soft tissue defects following a V-Y closure of the flap donor sites. Although these flaps were based on the opposite twins, we relied on the fluorescein studies, which showed that the vascular pattern across the soft tissue connections would adequately support random vascular pattern flaps (16). Indeed, both flaps exhibited only marginal epidermolysis at the very tips, which healed quickly. This technique was proven successful, as the complete abdominal wall reconstruction was possible immediately in twin B and 3 months after the primary operation in twin A, with a favorable esthetic outcome of the reconstruction.

A successful separation of CT requires delicate teamwork of experienced individuals in a number of specialties available only in a tertiary referral center. The current management of CT favors delayed separation, usually at the age of 6-12 months, which allows a thorough evaluation of anatomy and meticulously planned operation. However, the separation timing in omphalopagus twins is dictated by the specificities of physiology arising from the uniqueness of anatomy. We believe that extrauterine TTTS is a potential complication in CT that warrants urgent separation to avoid complications arising from significant circulatory crossover.
